# Rare and Low Frequency Variant Stratification in the UK Population: Description and Impact on Association Tests

**DOI:** 10.1371/journal.pone.0046519

**Published:** 2012-10-05

**Authors:** Marie-Claude Babron, Marie de Tayrac, Douglas N. Rutledge, Eleftheria Zeggini, Emmanuelle Génin

**Affiliations:** 1 Inserm UMRS-946, Genetic variability and human diseases, Paris, France; 2 Institut Universitaire d'Hématologie, Univ Paris Diderot, Paris, France; 3 AgroParisTech, Paris, France; 4 Wellcome Trust Sanger Institute, Hinxton, Cambridge, United Kingdom; Vanderbilt University, United States of America

## Abstract

Although variations in allele frequencies at common SNPs have been extensively studied in different populations, little is known about the stratification of rare variants and its impact on association tests. In this paper, we used Affymetrix 500K genotype data from the WTCCC to investigate if variants in three different frequency categories (below 1%, between 1 and 5%, above 5%) show different stratification patterns in the UK population. We found that these patterns are indeed different. The top principal component extracted from the rare variant category shows poor correlations with any principal component or combination of principal components from the low frequency or common variant categories. These results could suggest that a suitable solution to avoid false positive association due to population stratification would involve adjusting for the respective PCs when testing for variants in different allele frequency categories. However, we found this was not the case both on type 2 diabetes data and on simulated data. Indeed, adjusting rare variant association tests on PCs derived from rare variants does no better to correct for population stratification than adjusting on PCs derived from more common variants. Mixed models perform slightly better for low frequency variants than PC based adjustments but less well for the rarest variants. These results call for the need of new methodological developments specifically devoted to address rare variant stratification issues in association tests.

## Introduction

Population stratification is an important cause of false positive results in case-control association studies. Indeed, if the sampled population includes subgroups with different allele frequencies at some markers and different disease prevalences, there is a risk of detecting false positive associations between these stratified markers and the disease. This often occurs when the individuals are sampled from different ethnic groups. The most famous example in the literature is the association between a Gm haplotype and type 2 diabetes explained by Pima-Papago ancestry [1]. With the development of large scale genome wide association studies (GWAS), it was shown that the problem may even exist between populations that were previously assumed to be homogeneous such as Europeans [2], [3], [4], [5], [6], [7], [8], [9], [10]. Even between different regions of the United Kingdom (UK), important differences in allele frequency exist for some Single Nucleotide Polymorphisms (SNPs) such as those located on chromosome 2q21 in the region of the lactase gene that was shown to be under strong recent positive selection [11] or on chromosome 6p21 in the HLA region [12]. Thus, accounting for population stratification in GWAS is a crucial issue, leading researchers to reactualize methods proposed in the context of candidate gene studies [13], [14], [15], [16], [17] or to develop new methods to detect and correct for population stratification in case-control data [18], [19], [20], [21], [22], [23] (for a review see [24]). Simulation studies have shown that most of these methods perform generally well under different scenarios of population stratification [25] with usually a small advantage to the principal component corrected method, as implemented in the quick and simple Eigenstrat software [18]. More recently, mixed models that explicitly model population structure, family structure and cryptic relatedness have also been applied to GWAS and shown to perform even better than principal component analysis in situations where some individuals in the sample are related [26], [27], [28].

However, most of the studies on population stratification have focused on common genetic variants, namely SNPs with a minor allele frequency (MAF) above 5% because of the “common disease-common variant hypothesis” [29], [30]. More recently, after several GWAS have shown that common variants only explain a minor part of the heritability of most complex diseases, interest has moved to variants with lower frequencies. Rare variants have been shown to be involved in several diseases [31], [32], [33], [34], [35]. The hitherto identified rare risk variants often have functional consequences with a direct impact on protein functionality and tend to confer a stronger increase in disease risk than common variants. Rare variants are more likely to be under moderate levels of negative selection [36], [37] and/or to have arisen recently and be population-specific. Because of these differences between rare and common variants, it is plausible that rare variants could exhibit stratification patterns different from common variants. However, to date, the issue of rare variant stratification has not been addressed in the literature using real data except in the study of Heath et al. [5] where axes of variations in 13 European populations obtained using low frequency variants were strongly correlated to those obtained with common variants. This observation indicates that rare variants were not stratified differently from common ones in Europe. However, the conclusions drawn were limited because rare variants were very poorly represented in the SNP-chip used (Illumina 317K) and thus the study was underpowered to show differences between rare and common variants.

Our aim is to assess population stratification based on rare variants using the population-based control data from the WTCCC1 study and to examine how this stratification can impact the results of association tests, using the example of type 2 diabetes. We take advantage here of the high content in rare variants of the Affymetrix 500K SNP-chip used in the WTCCC1 study. This chip contains about 55,000 variants with MAF below 0.05 in Europeans, whereas the Illumina 317k chip contained approximately 9,000.

## Results

### Quality Control and MAF categories

**Figure 1:**
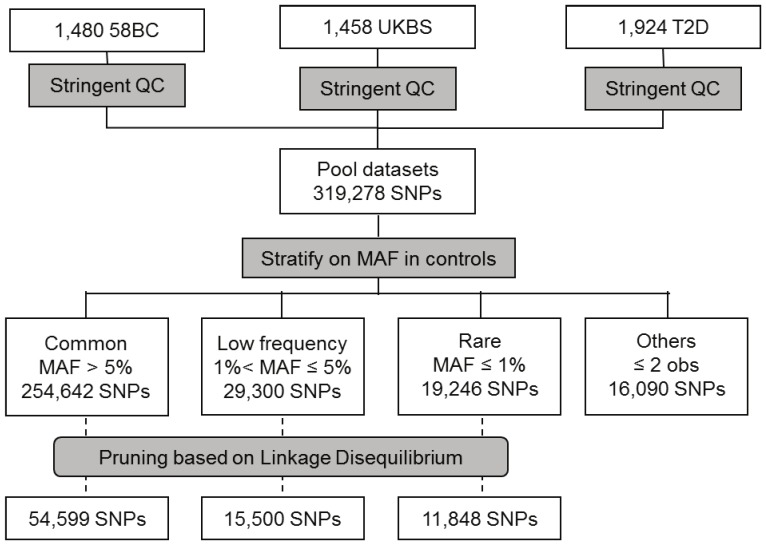
Flowchart showing the different QC steps and the number of SNPs in the different MAF categories.

Three sample sets from the WTCCC1 study (http://www.ebi.ac.uk/ega/home) are considered: the two control datasets (58BC with 1,480 individuals and UKBS with 1,458 individuals) and the type 2 diabetes (T2D) case dataset (1,924 individuals). These samples originate from 12 UK regions (see Table S1 and Figure S1) and are all genotyped on the Affymetrix 500K chip. After stringent quality control (QC) (see Methods), a total of 319,278 SNPs are available and classified into 4 categories depending on their minor allele frequency (MAF) in the total control group (58BC and UKBS combined) (Figure 1). A total of 254,642 (79.8%) SNPs with a MAF ≥0.05 are classified as “common”, 29,300 (9.2%) with a MAF in the range [0.01; 0.05] as “low frequency”, 19,246 (6%) with a MAF ≤0.01 but with a minor allele present in more than two copies in the combined set of controls are classified as “rare” and the last 16,090 (5%) with a minor allele present in 2 or less copies are classified as “others”(Table S2).

### Patterns of stratification in the control datasets

The two sets of controls have very similar allele frequencies at the different SNPs whatever the MAF category (Table S3 and Methods) except for the “others” category where an inflation of the genomic lambda is observed. The “others” category that includes SNPs with a minor allele seen only once or twice in the control datasets is more prone to genotyping assignment problems and will therefore no longer be considered in subsequent analyses.

**Figure 2:**
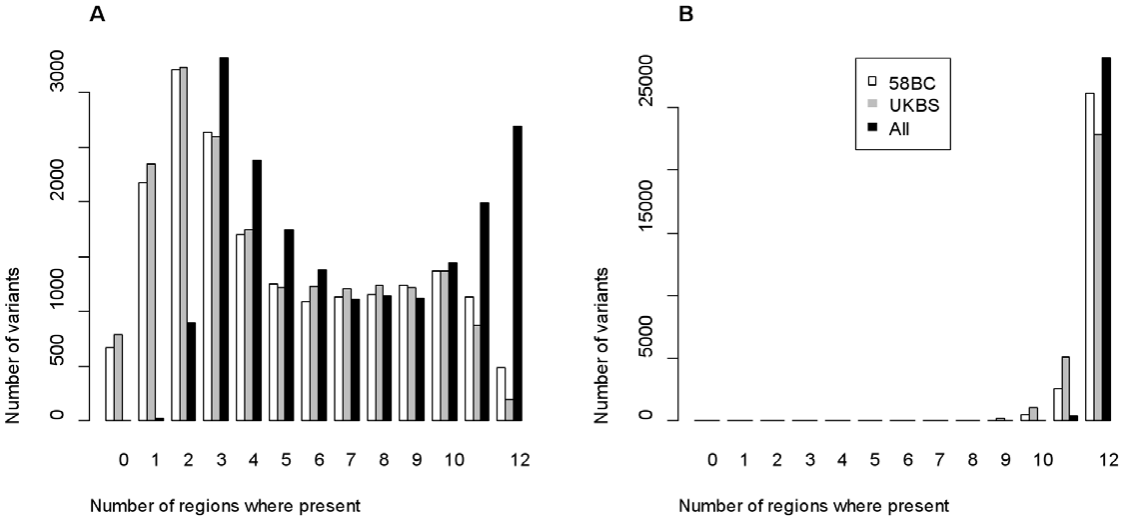
Distribution of the number of rare (A) and low frequency (B) variants shared by several regions.

On the pooled set of controls, an individual carries on average 110 rare variant alleles but this number varies between 48 and 563 with 95% of the individuals carrying less than 156 rare alleles. For low-frequency variants, these numbers are higher with an individual carrying on average 1,701 low frequency alleles with a range varying between 1,443 and 2,431 (Figure S2). The number of rare variants carried by each individual varies significantly between regions (p-value of the Kruskal-Wallis test = 1.8 10^−9^)(Figure S3). In particular, individuals from region R12 have less rare variants (mean and median numbers per individual are 101.6 and 97, respectively) than individuals from the other regions, especially in regions R1, R2 and R4 (mean and median number of rare variants per individual are, respectively, 115.5 and 109 for R1, 114.0 and 108 for R2, 115.3 and 108 for R4). Differences in allele frequencies between the 12 UK regions show similar patterns over the different MAF categories with similar proportions of SNPs strongly differentiated between regions (Table S4). Most of the rare variants are polymorphic in several regions and about 14% (i.e., 2,693 out of the 19,246) are even found in all 12 regions (Figure 2A). Interestingly, 14% is exactly the proportion of rare variants expected to be shared by the 12 UK regions if rare variants were homogeneously distributed over the UK regions. In contrast, a majority of the low frequency variants (more than 98%) are found in all regions (Figure 2B). These patterns of regional distribution of rare and low frequency variants are very similar in the two control datasets. However, a large proportion of the rare variants unique to one region in one of the control datasets are spread over several regions in the other control dataset. This probably illustrates the fact that the rare variants present on the chip are slightly biased towards higher frequencies. Moreover, only 18 (8.2%) of the 220 variants unique to one region in both the UKBS and 58BC samples are found in the same region in these two samples (Table S5).

**Figure 3:**
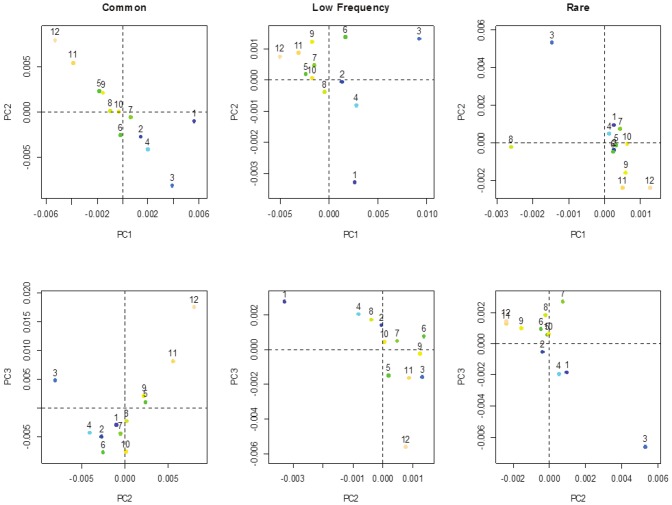
PCA for the different pruned MAF sets. The mean of PC1 and PC2 scores (top row) and of PC2 and PC3 scores (bottom row) in each region are plotted considering the common variants, the low frequency variants and the rare variants.

The distributions of the principal component (PC) scores for individuals from the different UK regions overlap considerably. No difference in these scores is found when comparing the two control datasets, ruling out concerns of batch effects between them (data not shown). However, significant differences are observed between the different regions for the top PC computed on the 3 MAF sets (Figure S4 and Table S6). To investigate geographic population structure, the means of the top 3 scores of individuals within a region were calculated and plotted in Figure 3. These PCs are informative about regional geographical structure but the positions of the different regions differ depending on the pruned MAF set considered. When only the common variants are included in the analysis, the regions tend to separate more on PC1, PC2 and PC3 than when low-frequency or rare variants are considered. Note that the plot of PC3 against PC2 obtained for common variants is very similar to the one reported in [38]. The screeplots (Figure S5) show that the percentage of variance explained by the top PCs is much greater for rare variants (∼35% for PC1 and ∼28% for PC2) than for low frequency (∼9% for PC1 and ∼8% for PC2) or common variants (∼7% for both PC1 and PC2) suggesting that the rare variant stratification is stronger than the one of more common variants. Common, low-frequency and rare SNPs tell different stories regarding population stratification, as can be seen from the very low levels of correlations (R^2^) between PCs. PCs computed from the rare variant set are poorly correlated to the PCs computed from the common or low frequency sets and this, even if we consider jointly several PCs extracted from these different sets and look if one PC from one set is captured by several PCs from another set (Table 1). This is especially striking for PC1 from rare variants that is not captured at all by the top 10 PCs from common variants (R^2^ = 0.02). To rule out the possibility that this could be due to stochastic variations given the low number of SNPs used to perform the PCA (11,848 SNPs for the rare variant set after pruning), we extracted from the 54,599 pruned common SNPS, four disjoint subsets of 11,848 SNPs and measured their correlations (Table S7). We found that such a low number of SNPs is not sufficient to fully capture the subtle population structure effects within the UK as the correlations between the PCs extracted from these four different subsets are far from 100% with R^2^ values between the PC1 from the different sets ranging from 0.26 to 0.33. However, these correlation values are much higher than the ones observed between PC1 computed from the rare variant set and the top 10 PCs computed from the common variants (R^2^∼0.30 compared to 0.02). This is less true for PC2 from rare variants that shows a correlation with PC2 from common variants of 0.28, similar to the level of correlation observed on the disjoint subsets of common SNPs. PCs computed using the low frequency variant set show R^2^ correlation values of about 60% with the top 10 PCs from both the rare and common variants sets, showing that their stratification pattern is relatively well captured by the PCA performed on either set of variants.

**Table 1:** Correlation (R^2^ values) between the first two PCs (PC1.x and PC2.x) obtained on the different subsets of variants (x = “common”, “lowfreq” or “rare”).

	PC1.common	PC2.common	PC1.lowfreq	PC2.lowfreq	PC1.rare	PC2.rare	Top10.common	Top10.lowfreq	Top10.rare
**PC1.common**	1.00	0.00	0.14	0.44	0.00	0.07	1.00	0.59	0.13
**PC2.common**		1.00	0.50	0.12	0.01	0.19	1.00	0.63	0.43
**PC1.lowfreq**			1.00	0.00	0.03	0.31	0.67	1.00	0.65
**PC2.lowfreq**				1.00	0.00	0.00	0.57	1.00	0.00
**PC1.rare**					1.00	0.00	0.02	0.04	1.00
**PC2.rare**						1.00	0.28	0.31	1.00

The last three columns Top10.x give the cumulative R^2^ values over the top 10 PCs to show how each PC.x in line is captured by the combined top 10 PCs of the different subsets.

Although the top PCs of the PCA performed on the different pruned MAF sets appear to be strongly correlated to region of origin (as shown by the extreme P-value of the ANOVA comparing PC values per region on Table S6), supervised analysis with Admixture (see Methods) fails to correctly assign individuals to their reported geographic region of origin. For most of the individuals, there are strong ambiguities regarding the regions they belong to (Figure S6) and the estimated posterior probabilities to belong to their true region are very low (Figure S7). The different sets of variants give similar results, although common variants perform slightly better. Indeed, among the 1,458 UKBS controls, the number of individuals assigned to their correct region with a posterior probability greater than 50% are respectively 166 (11.39%), 102 (7.00%) and 132 (9.05%) for common, low frequency and rare variants. To determine if the better performances of Admixture on common variants could be explained by the fact that they outnumber the rare and low frequency variants, we also run Admixture on the disjoint subsets of 11,848 common SNPs and found that indeed the number of individuals assigned correctly to their region decreases (119 to 139 depending on the subset; i.e. 8.16% to 9.53%). The proportions of correctly assigned individuals also vary between regions, with regions R11 (Wales) and R12 (Scotland) showing better performance than the others. This could probably be explained by their more isolated geographic locations but it is also possible that the recorded regions of origin are not an accurate reflection of ancestry within the UK.

### Impact of the stratification of the different sets of variants on association tests

Geographical structure in UK datasets, if not well accounted for, could lead to an inflation of the number of false positive associations. This inflation is obvious for rare variants in the simulated scenario of strong stratification where a larger proportion of individuals from region R12 are assumed to be cases (0.55 from region R12 versus 0.40 from region R11) and compared to control individuals more often sampled in region R11. The genomic control lambda values λ_GC_ of the tests without correction are then respectively 1.006, 1.058 and 2.1928 for the common, low frequency and rare categories (Table 2). When the rare variant category is further subdivided into very rare (MAF≤0.005) and less rare (MAF>0.005), we see that the inflation is larger for the rarest variants with respective λ_GC_ for these two categories of 2.193 and 1.121 for the unadjusted test. As expected, the Cochran-Mantel-Haenszel (CMH) test that accounts well for the bias introduced by stratifying on the 2 regions shows λ_GC_ values closer to one on the different sets of variants even if these λ_GC_ values still remain slightly inflated for the low frequency variants (λ_GC_ = 1.023) and more strongly inflated for the rare variants (1.802 for the entire set and 1.103 for the one with MAF >0.005). When the tests are adjusted on the PCs, λ_GC_ values are decreased compared to the unadjusted tests and tend towards those observed for the CMH test. When adjustment is performed on 10 PCs, the λ_GC_ values are slightly smaller than those of the CMH and this might indicate some over-adjustment. No major differences are seen whether the adjustment is performed with PCs computed using the pruned MAF sets corresponding to the tested variant or not. Moreover, the mixed model implemented in EMMAX provides good results for the common category but leads to inflation factors very similar to the uncorrected test for low frequency and rare variants and thus slightly worse than the PC-based corrections. In the more realistic scenario where association with type 2 diabetes is tested, we also observe that the λ_GC_ values of PC adjusted tests are very similar when PCs are computed on variants with the same allele frequencies as the ones tested or on variants with different frequencies (Table S8). Focusing on rare variants, we see that the most extreme p-values are found in the same regions with the different tests and are very similar to the p-values of the unadjusted test except for the CMH test with p-values one order of magnitude smaller (Figure S8).

**Table 2:** Genomic control coefficient lambda λ_GC_ obtained for the different tests of association performed on the simulated scenario of stratification in regions R11 and R12.

				Within the Rare set	
	Common	LowFreq	Rare	MAF< = 0.005	MAF>0.005
**CMH** a	0.993	1.023	1.802	1.802	1.103
**Raw** b	1.006	1.058	2.193	2.193	1.121
**common.2** c	0.993	1.042	2.072	2.137	1.067
**common.3**	0.993	1.042	2.052	2.129	1.066
**common.4**	0.990	1.048	1.927	2.053	1.104
**common.10**	0.978	1.027	1.675	1.880	1.100
**lowfreq.2**	0.995	1.044	1.969	2.085	1.072
**lowfreq.3**	0.995	1.041	1.945	2.044	1.064
**lowfreq.4**	0.992	1.033	1.922	2.022	1.071
**lowfreq.10**	0.980	1.012	1.767	1.931	1.112
**rare.2**	0.993	1.040	2.085	2.152	1.089
**rare.3**	0.991	1.043	2.081	2.143	1.090
**rare.4**	0.989	1.032	1.960	2.074	1.081
**rare.10**	0.974	1.014	1.858	2.013	1.072
**EMMAX.common** d	1.001	1.052	2.182	2.182	1.115
**EMMAX.lowfreq**	1.001	1.052	2.182	2.182	1.115
**EMMAX.rare**	1.000	1.048	2.165	2.180	1.114

a Cochran-Mantel-Haenszel test accounting for the 2 different regions.

b Test not corrected for population stratification.

c Test corrected for population stratification using different number of PCs computed on different pruned MAF sets (i.e.; common.2 means that the PCs were computed on the common varint sets and 2 the test is adjusted on 2 such PCs).

d Test performed using the mixed model implemented in EMMAX with the relatedness matrix computed either on the common, low frequency or rare variant sets.

## Discussion

With the development of next-generation sequencing technologies, investigators will soon be able to study the impact of rare variants on complex traits. Methods have been developed to test for association between rare variants and disease in case-control datasets: for reviews, see [39], [40]. Most of these methods assume that samples are homogeneous and not stratified in subpopulations. Previous studies on common variants have shown that such an assumption is unrealistic and that it is necessary to properly account for population stratification in case-control association tests to avoid false positive findings [9], [41]. If the population stratification of common variants has been extensively studied on empirical data, this is not the case for rare variants. Intuitively, one would expect the stratification issue to be more problematic for rare variants than for more common ones as these rare variants are more likely to cluster in particular geographic regions. Using the WTCCC1 control data, we show that indeed several rare variants are only found in one or a few of the 12 UK regions and that there is no strong correlation between the axes of variation of allele frequencies observed in the UK for rare and more common variants.

Indeed, when performing a principal component analysis on the different sets of variants determined based on their MAF, we found that the top PCs are poorly correlated. This result is in discrepancy with the one reported by Heath et al. [5] using data on 5,847 individuals originating from 13 countries across Europe. In their study, the authors compared the top PCs obtained when principal component analyses were performed using a panel of 8,412 low frequency SNPs with a MAF of less than 5% and using a panel of 8,734 common SNPs with a MAF greater than 0.485. They found that these two panels of markers gave the same overall pictures with the first two PCs showing very strong correlations above 0.8. This is not the case in our data where individual PCs computed on the different pruned MAF sets are poorly correlated. Differences between our study and that of Heath et al. [5] could first be explained by the fact that the SNPs studied are different and the Affymetrix 500K SNP-chip used here is more enriched in rare variants than the Illumina 317k used by Heath et al. The populations studied are also different and so is the extent of genetic variation in these populations. Indeed, there are more variations at the scale of the entire Europe than at the scale of the UK and the top PCs on the European data explain more of this variation than our top PCs. If individual PCs computed on one set of variants were poorly correlated to individual PCs computed on another set, some stronger correlations were observed when considering several PCs together. This was true especially for the low frequency set since PCs from this set were well explained by a combination of several PCs from the common or the rare variant sets. On the other hand, results were not improved for PC1 from rare variants that was not explained by any combination of PCs extracted from common or low frequency variants. Since this PC1 explains much more variance than the first few PCs extracted from the other datasets and than PC2 from the rare variants, we believe it could in fact capture an important axe of variation of rare variants, although we cannot exclude this might be due to some genotyping problems or batch effects we tried to avoid as much as possible by using very stringent QC criteria. It is also interesting to note that the PC1 values from rare variants were not differentially distributed over the different regions, indicating that this component captures a stratification that is not well correlated with the UK regions. However, it is also possible that part of the effect could in fact be due to the fact that, in the PCA, an allele frequency weighted coding was used for each SNP that assigns higher weights to rare variants and that could inflate genotyping error effects of rare variants. Indeed, when the analysis was redone without allele frequency weighting, we found that the first two PCs were inverted with PC1 being now differentially distributed over the geographic regions and PC2 no longer showing this differentiation.

Since the top PC values calculated on the different sets of markers are all significantly different between the 12 UK regions, except for PC1 from rare variants, one could wonder whether it would be efficient to use principal component analysis to cluster individuals and then perform stratified analyses to test for association. Such a strategy for association testing where individuals are first clustered using the genetic information and then stratified association tests are performed was first proposed by Pritchard et al. [42] using the efficient model-based clustering method implemented in STRUCTURE [15]. More recently, several other clustering methods based on distances computed from genetic data [21], [22], [43], [44], [45], [46] have been proposed. To determine if such a strategy could indeed be useful when testing for association with lower frequency variants, we used Admixture to determine how well the region of origin of the controls could be inferred from the SNP data. We found that overall the predictions are not very good, but that they are similar whatever the MAF category. This could be due to the fact that the reported regions of origin are not very accurate or it could indicate that the SNP data are not informative enough to clearly cluster individuals within the UK. Indeed, a single cluster is the best solution when running Admixture on the whole panel of controls (data not shown).

Results on type 2 diabetes also tell us that it is not easy to adjust for stratification in rare variant association tests. Since the PCs computed on common and rare variants were poorly correlated, one would have expected to see an improvement when adjusting on PCs computed from rare variants as compared to PCs computed from common variants. This was not the case however even when the level of stratification was strong as in the extreme stratification scenario that we simulated. Interestingly, mixed models did also no better than simple PC-based corrections for rare variants. Since these models have been shown to better adjust for stratification of common variants, especially in the presence of remote relatedness between individuals, one could have expected an improvement for rare variants. Indeed, remote relatedness might be a concern when studying rare variants since carriers of a same rare allele are likely to be more related than random individuals. Recently, Mathieson and McVean [47] reached similar conclusions regarding the difficulty to adjust for rare variant stratification in association tests using existing methods. Their results combined to ours emphasize the need for methodological developments to better account for rare variant stratification patterns in association tests. It should be noted however that in our study we did not explore the impact of population stratification on gene-based variant collapsing methods that are often used to test for association with rare variants. It is possible that these methods could be more robust to stratification but we could not study them as the number of rare variants within each gene present on the SNP-chip was too small.

A major concern in our study is the fact that the rare variants we considered here might not be very representative of rare variants that would be discovered through resequencing studies. Indeed, to be included in the SNP-chip, these variants need to have been observed on other samples than the one investigated here and we are therefore not investigating the lower tail allele frequency spectrum. The number of rare variants investigated is also much reduced as compared to what will be found in resequencing studies and the investigation of population stratification based on such a small number of variants might not be very reliable. We could also not exclude the possibility that genotyping errors could have obscured regional membership assignment especially for the rare variants that are more prone to genotyping errors than more common ones. The current dataset was not ideal to answer the question raised and our observations will thus need to be confirmed on other more appropriate datasets. With the increasing availability of sequence data, it will soon be possible to perform a study similar to ours using rare variants discovered through direct resequencing. Nevertheless, we believe it is important to evaluate rare variant stratification at this stage and using the available empirical data to guide researchers in the planning of their future studies using next-generation sequencing technologies.

## Methods

### Quality Control

Stringent quality control (QC) was performed in several steps. First, each dataset was treated separately and only SNPs with an rs number and genotypes with a calling probability >0.99 were kept. Second, SNPs were eliminated if their call rates were lower than 0.99 in any of the three samples or if their genotype distribution in the two control datasets was significantly different from the one expected under Hardy-Weinberg proportions (p-value<10^−8^). Finally, only the SNPs retained in all three samples were kept, giving a total of 319,276 SNPs post-QC. No specific QC was performed on the individuals as the 4,862 individuals considered here were the ones selected in the WTCCC1 study after QC.

### Patterns of stratification in the control datasets

Homogeneity between the two sets of controls (58BC and UKBS) was first tested for each MAF category using a 1 degree of freedom (d.f.) Mantel-extension test of the difference in MAF between subjects from the 58BC and UKBS collections, stratified by the 12 broad regions of the United Kingdom. Homogeneity of the two control datasets was assessed by the Genomic Control lambda [13], λ_GC_, which is the ratio of the median of the observed chi-square values over the expected median of a 1-df chi-square. To study the stratification between the 12 UK regions, the two sets of controls were pooled and homogeneity between the 12 regions was tested using an 11-df chi-square test. Significance was evaluated by permutations as implemented in Plink [48](http://pngu.mgh.harvard.edu/~purcell/plink/). In the absence of heterogeneity, the observed p-values are uniformly distributed, and the quantity −2*log(observed p-value) follows a 2-df chi-squared [49]. We therefore introduced a measure of overdispersion (referred to as genomic inflation coefficient) for each MAF category, λ_r_. Similarly to the Genomic Control lambda [13], λ_r_ is the ratio of the median of −2*log(observed p-value) over the expected median of a 2df chi-squared distribution.

In a next step, the SNPs in each MAF category (“common”, “low frequency” and “rare”) were first pruned on Linkage Disequilibrium (Plink options: “indep-pairwise 50 5 0.2”). Principal component analysis of the genotype data using SmartPCA from the Eigensoft package [18], [50](http://genepath.med.harvard.edu/~reich/EIGENSTRAT.htm), was performed on the three pruned MAF sets and the correlation between the top principal components (PC) obtained in these different analyses were computed.

To determine how much of the regional geographic information can be inferred from the genetic data, the Admixture software [51] (http://www.genetics.ucla.edu/software/admixture) was used to impute the region of the UKBS controls, assuming the region of origin of the 58BC controls was known. This supervised analysis was carried out for each MAF category with the same pruned MAF sets as for the PCA.

### Impact of the stratification of the different categories of variants on association tests

The impact of rare variant stratification on the results of case-control association studies was evaluated in an extreme scenario of stratification where cases and controls were sampled from regions R11 and R12 in different proportions. Among the 146 individuals from region R11, 59 (40.4%) were assigned cases and the remaining individuals were assigned controls and among the 273 individuals from region R12, 150 (54.9%) were assigned cases and the remaining individuals were assigned controls. First a Cochran-Mantel-Haenszel (CMH) test accounting for the 2 different regions (as implemented in Plink with p-values estimated by case-control status permutations within each stratum) was performed in each MAF category. Since this test adjusts exactly on the confounding factor introduced in the analysis, we used it as reference to assess the performances of the other tests. Second, the association test statistics in each MAF category were calculated either ignoring the stratification or after adjusting on the PCs derived from the PCA described above, using eigenstrat from the Eigensoft package [18], [50]. Third, the mixed model implemented in EMMAX [28] was used with relatedness matrix computed using the different sets of variants. Genomic control lambdas λ_GC_ were computed from the observed chi-squared distributions or after transforming the p values obtained from EMMAX into a chi-square with one degree of freedom.

Finally, a more realistic scenario where the type 2 diabetes cases were compared to all the controls was also investigated with the same methods except for the CMH test that was stratified on the12 regions instead of 2.

## Supporting Information

Figure S1 **Map of UK with the 12 regions of origin, as defined by the WTCC1 study (reprinted from ref. 12 with permission from the authors).**
(TIF)

Figure S2 **Distribution of the number of rare and low frequency variants carried by each individual in the combined WTCCC control datasets.**
(TIFF)

Figure S3 **Distribution of the number of rare variant per individual in the 12 UK region.**
(TIF)

Figure S4 **Boxplots of the PC values per region for the first 3 PCs computed on the different pruned MAF sets.**
(TIF)

Figure S5 **Percentage of variance explained by the different principal components from the principal component analysis performed on the different sets of markers.**
(TIF)

Figure S6 **Pie charts showing the average posterior probabilities of UKBS controls from each of the 12 regions to belong to any of the 12 regions.** A. when Admixture is run on the set of common variants, B. when Admixture is run on the set of low frequency variants and C. when Admixture is run on the set of rare variants.
(PDF)

Figure S7 **Posterior probabilities for the UKBS individuals to belong to their true region of origin P_true_ (in black) or another region P_other_ (in grey).** These posterior probabilities were computed with Admixture [51] using the different pruned MAF sets (common, low frequency and rare). The individuals are sorted by region and ranked by increasing values of P_true_ within each region.
(TIFF)

Figure S8 **Manhattan plots showing the association between T2D and the rare variants, detected without correction (“raw”), with the CMH test (“cmh”), after correction with the first 10PCs of the common (“PCA common”) and of the rare (“PCA Rare”) MAF sets, and after correction with Emmax on the common (“Emmax Common”) and the rare (“Emmax rare”) MAF sets.**
(TIFF)

Table S1 **Case and control repartition by region.**
(DOCX)

Table S2 **Description of the “others” SNPs according to the number of minor allele copies in the different samples.**
(DOCX)

Table S3 **Results** **of homogeneity tests performed to compare allele counts in the two subgroups of controls.** Genomic Control lambda values (λ_GC_) are provided for the different MAF categories.
(DOCX)

Table S4 **Number and proportion of variants that differ the most (p-value<5.10^−4^) between the 12 UK regions in the different MAF categories.**
(DOCX)

Table S5 **Repartition of the SNPs seen in only one region in each dataset, UKBS and 58BC.** SNPs unique to each of the 12 regions are compared for their regional distribution in the two datasets and those found in the same region in the two datasets are highlighted.
(DOCX)

Table S6 **P-values of the ANOVA comparing the values of the different PCs over the 12 region.**
(DOCX)

Table S7 **Correlation (R^2^ values) between the first two PCs (PC1.x and PC2.x) obtained on the four disjoint subset x (x = 1 to 4) of 11,848 common variants.** The last three columns Top10.x give the cumulative R^2^ values over the top 10 PCs to show how each PC in line is capture by the combined top 10 PC of the different subsets.
(DOCX)

Table S8 **Genomic control coefficient lambda λ_GC_ obtained for the different tests of association performed on the type 2 diabetes dataset with the different sets of variants.**
(DOCX)
