# Supplementary figures and images for: Rare and Low Frequency Variant Stratification in the UK Population: Description and Impact on Association Tests
Source: PLoS One. 2012 Oct 5;7(10):e46519. doi: 10.1371/journal.pone.0046519 (PMC3465327; doi:10.1371/journal.pone.0046519)

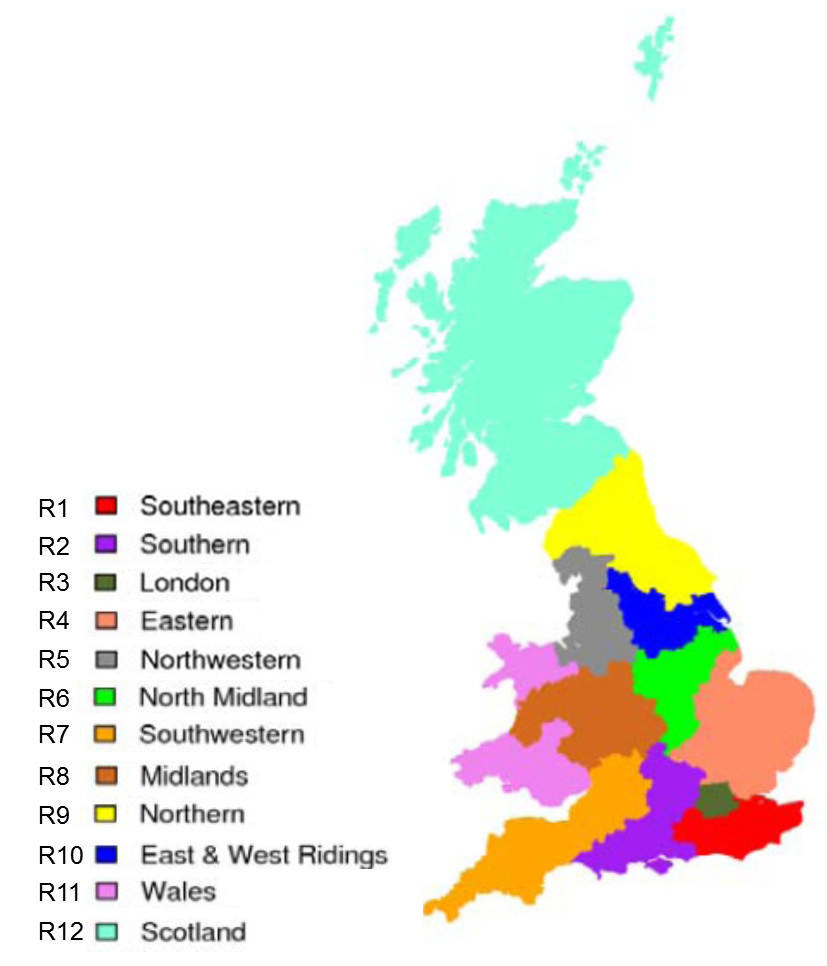

Supplement: Figure S1 — Map of UK with the 12 regions of origin, as defined by the WTCC1 study (reprinted from ref. 12 with permission from the authors). (TIF) [file pone.0046519.s001.tif]

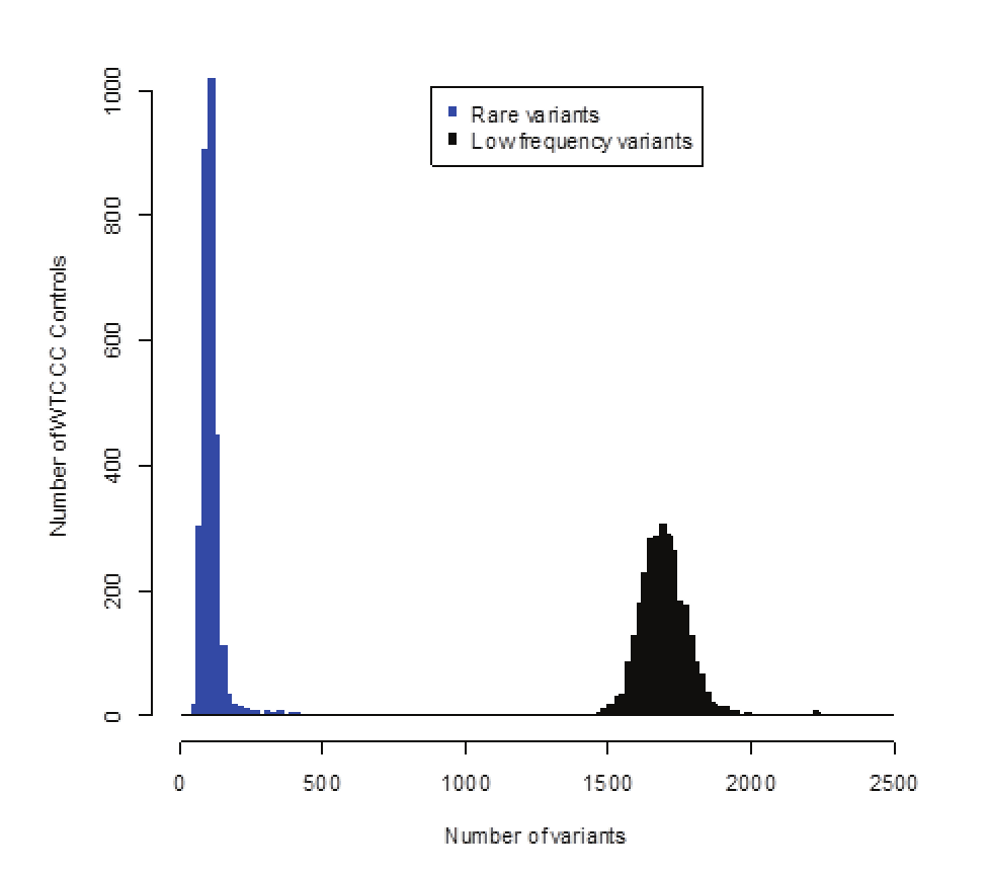

Supplement: Figure S2 — Distribution of the number of rare and low frequency variants carried by each individual in the combined WTCCC control datasets. (TIFF) [file pone.0046519.s002.tiff]

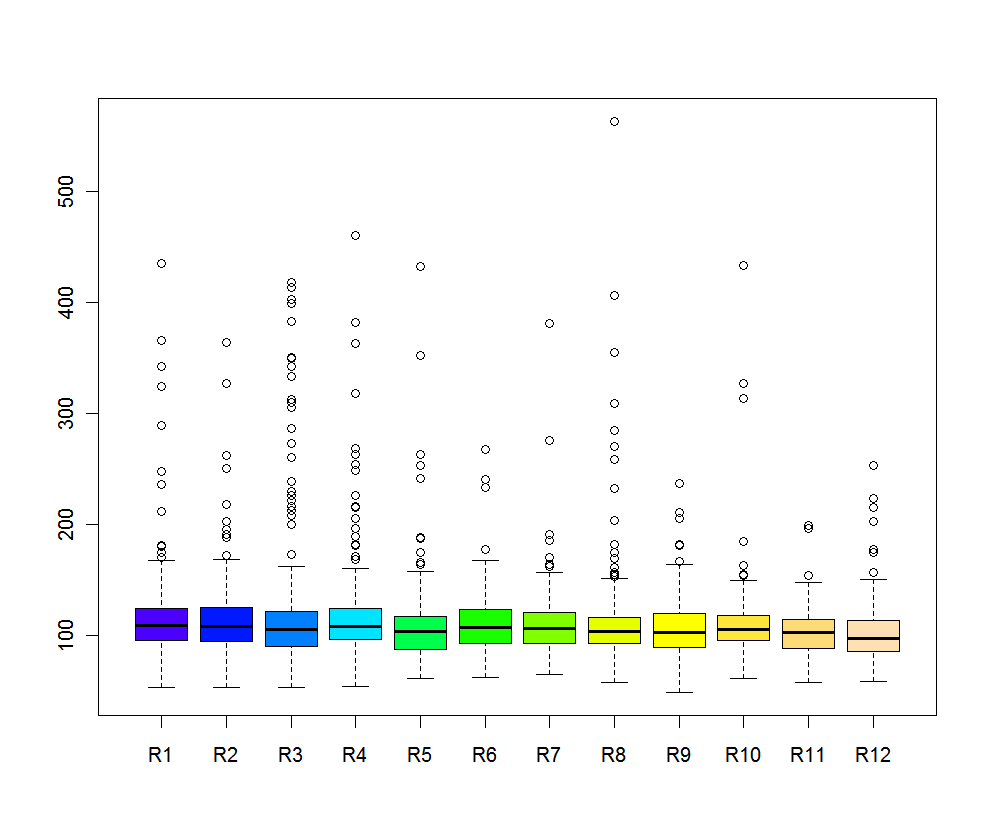

Supplement: Figure S3 — Distribution of the number of rare variant per individual in the 12 UK region. (TIF) [file pone.0046519.s003.tif]

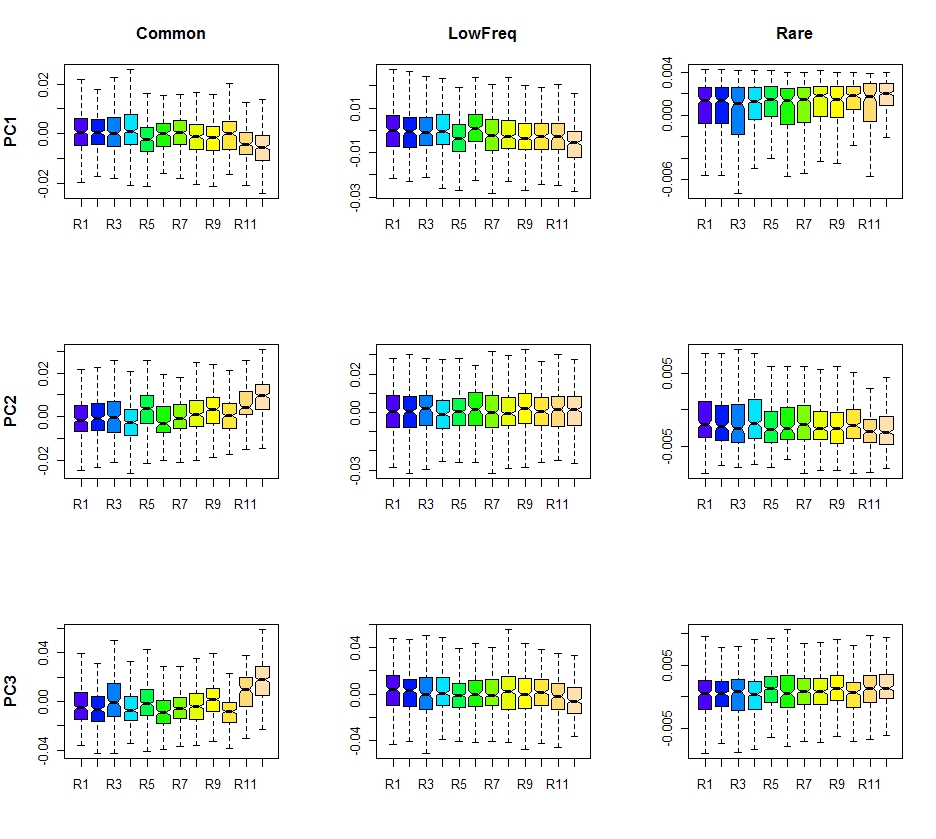

Supplement: Figure S4 — Boxplots of the PC values per region for the first 3 PCs computed on the different pruned MAF sets. (TIF) [file pone.0046519.s004.tif]

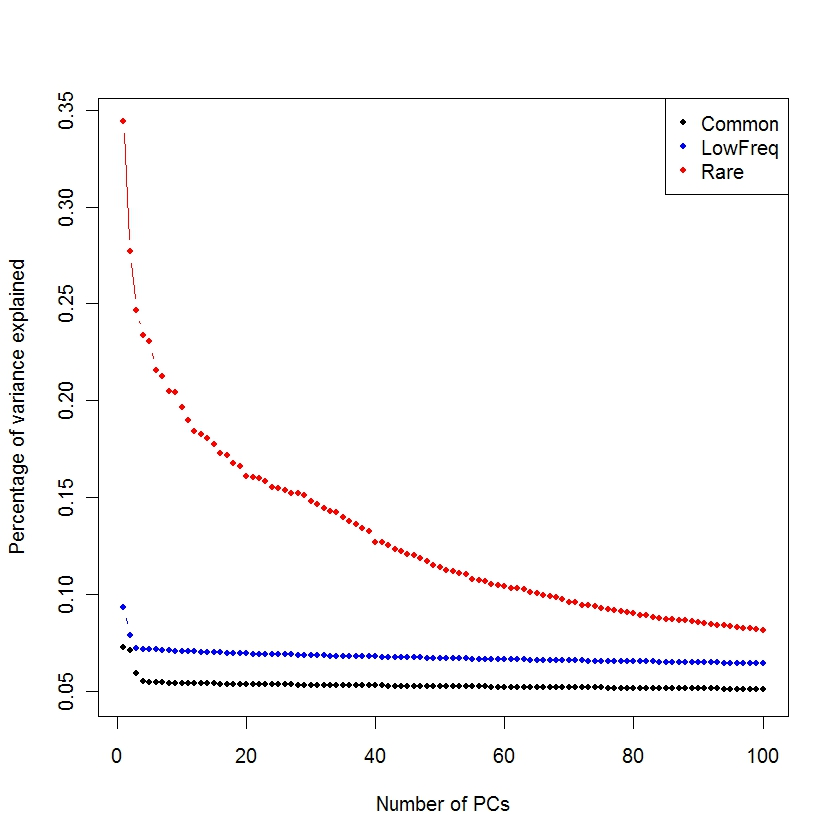

Supplement: Figure S5 — Percentage of variance explained by the different principal components from the principal component analysis performed on the different sets of markers. (TIF) [file pone.0046519.s005.tif]

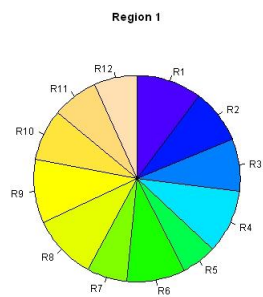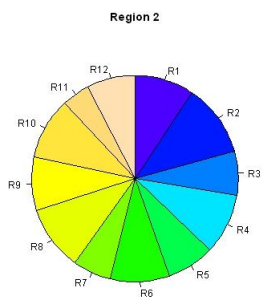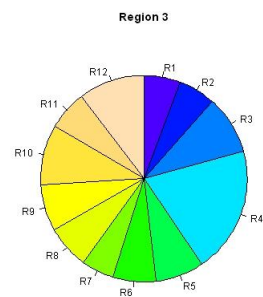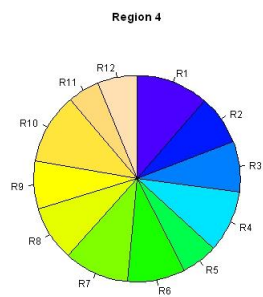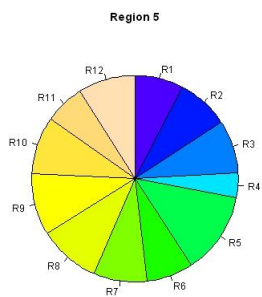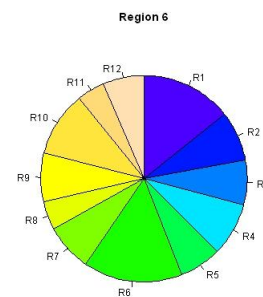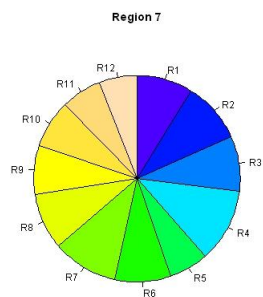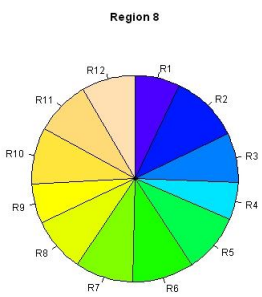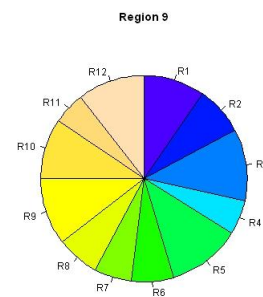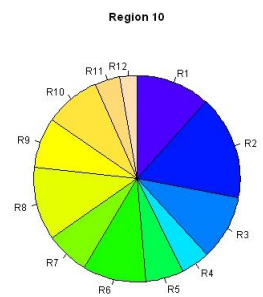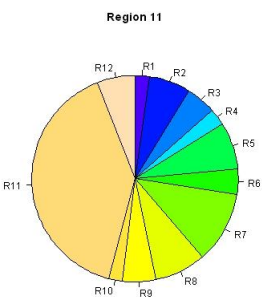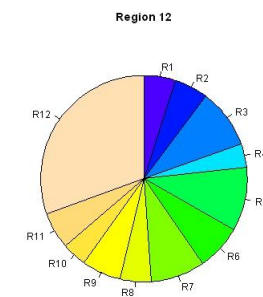

A

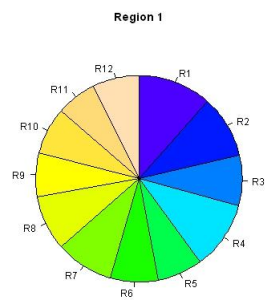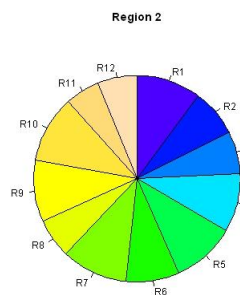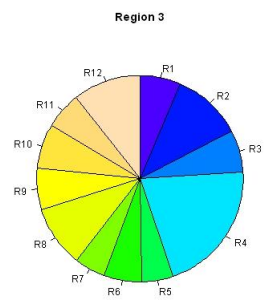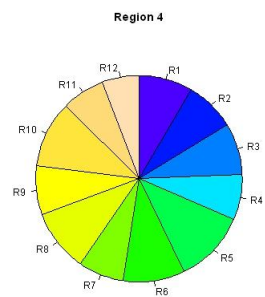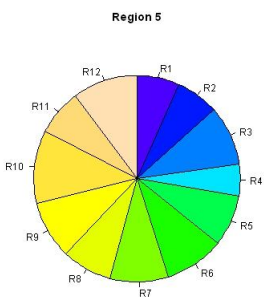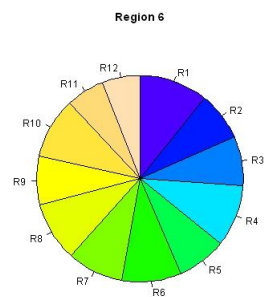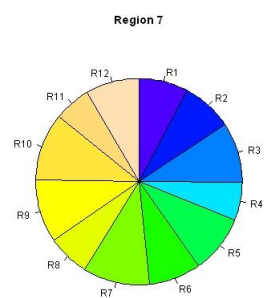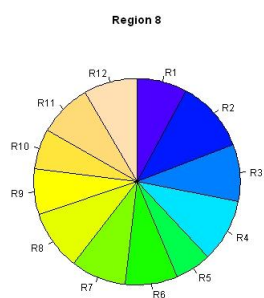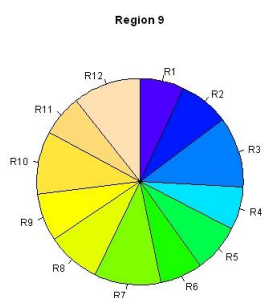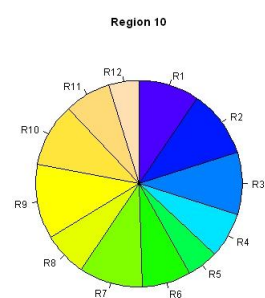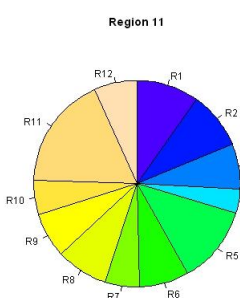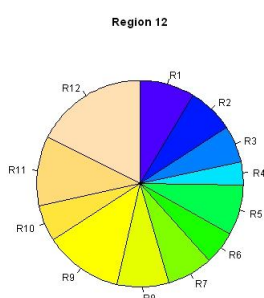

B

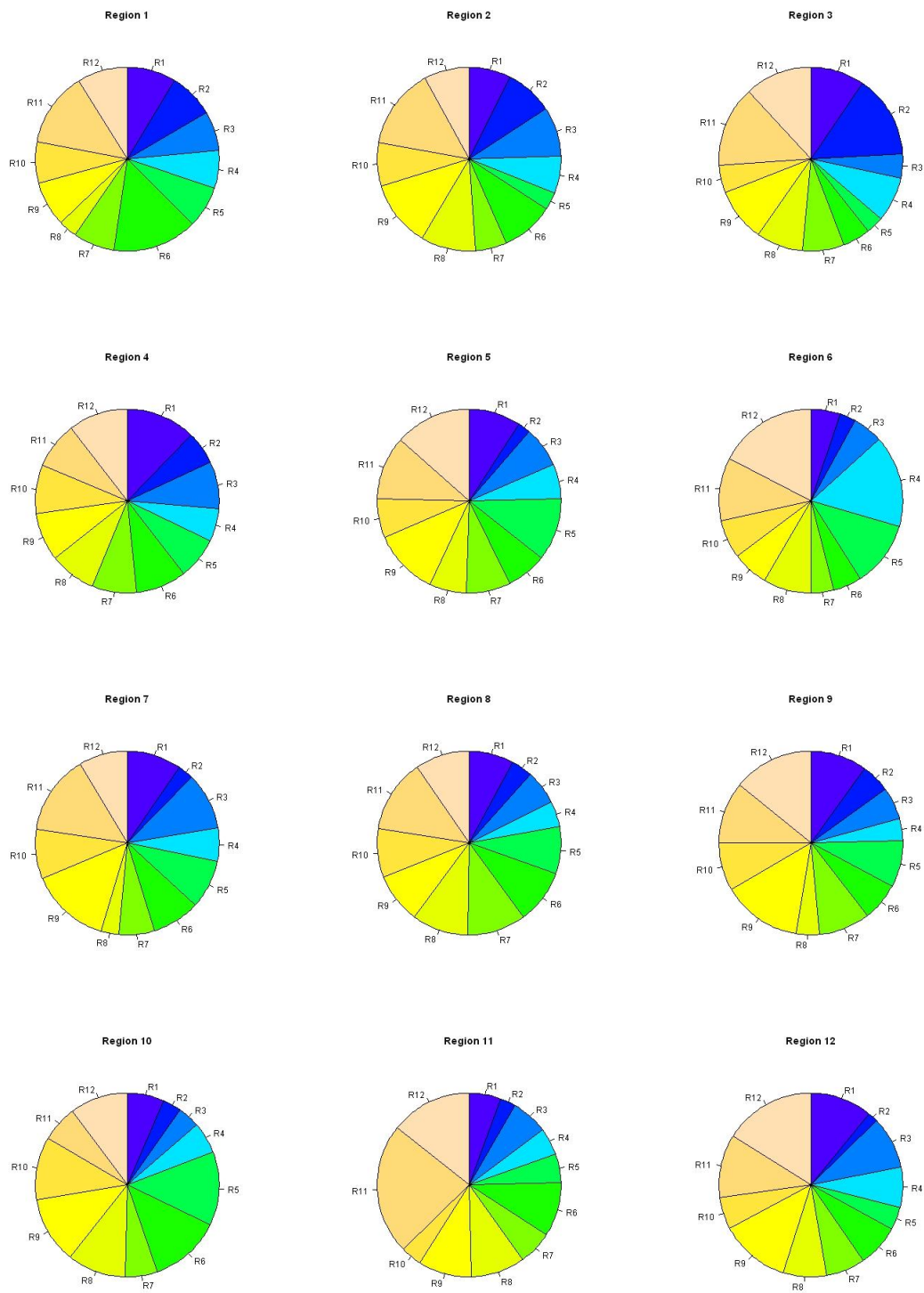

C

Supplement: Figure S6 — Pie charts showing the average posterior probabilities of UKBS controls from each of the 12 regions to belong to any of the 12 regions. A. when Admixture is run on the set of common variants, B. when Admixture is run on the set of low frequency variants and C. when Admixture is run on the set of rare variants. (PDF) [file pone.0046519.s006.pdf]

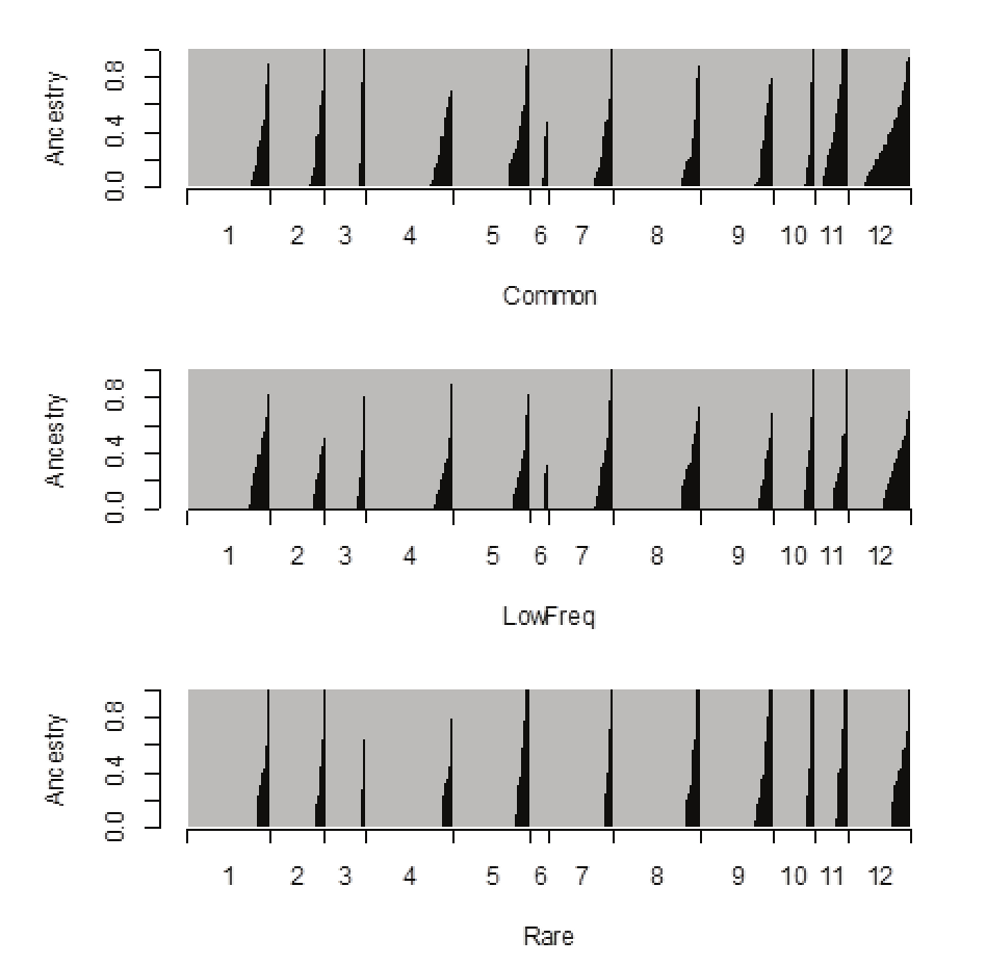

Supplement: Figure S7 — Posterior probabilities for the UKBS individuals to belong to their true region of origin Ptrue (in black) or another region Pother (in grey). These posterior probabilities were computed with Admixture [51] using the different pruned MAF sets (common, low frequency and rare). The individuals are sorted by region and ranked by increasing values of Ptrue within each region. (TIFF) [file pone.0046519.s007.tiff]

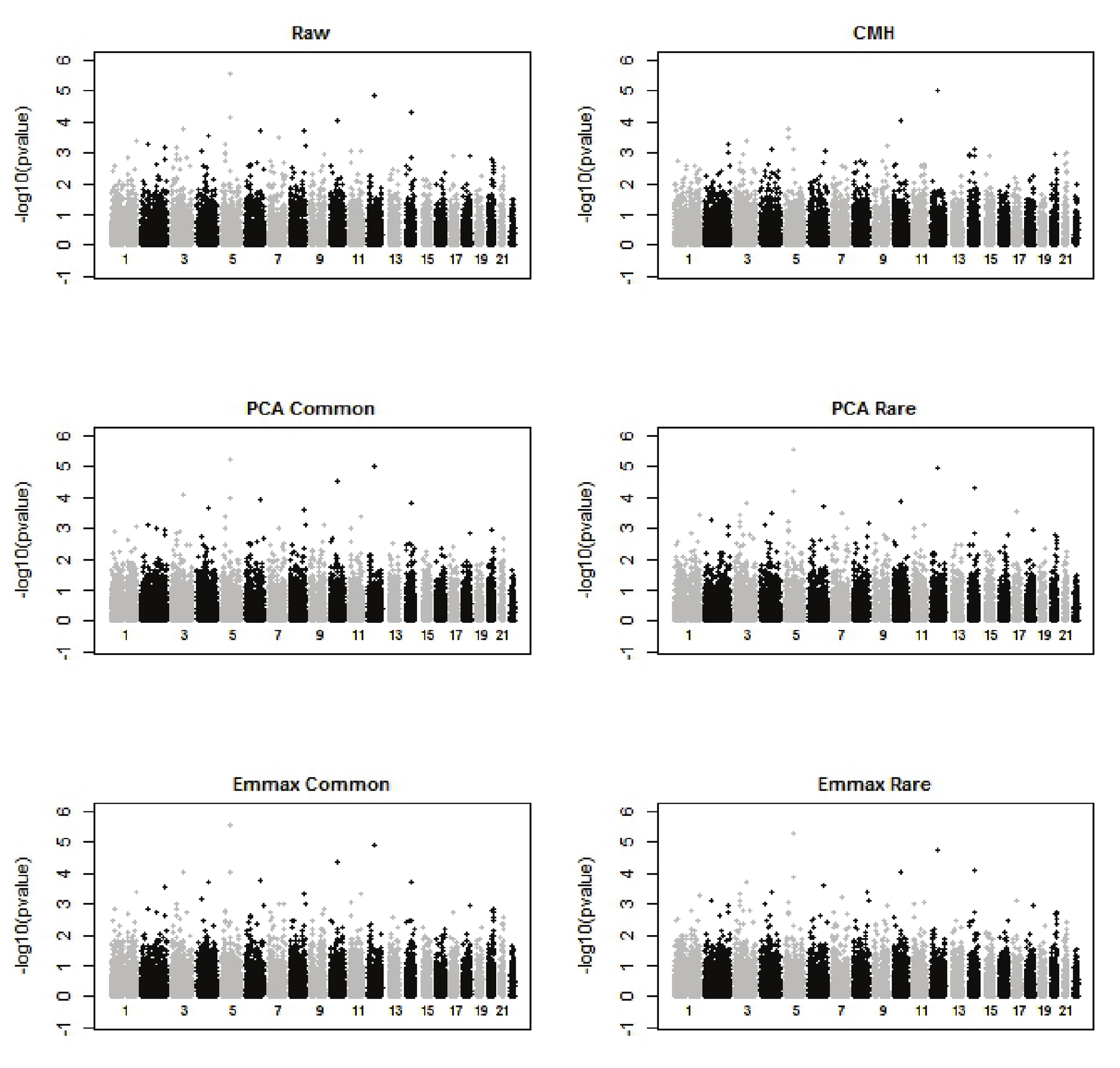

Supplement: Figure S8 — Manhattan plots showing the association between T2D and the rare variants, detected without correction (“raw”), with the CMH test (“cmh”), after correction with the first 10PCs of the common (“PCA common”) and of the rare (“PCA Rare”) MAF sets, and after correction with Emmax on the common (“Emmax Common”) and the rare (“Emmax rare”) MAF sets. (TIFF) [file pone.0046519.s008.tiff]
