# Supplementary material for: Rare and Low Frequency Variant Stratification in the UK Population: Description and Impact on Association Tests
Source: PLoS One. 2012 Oct 5;7(10):e46519. doi: 10.1371/journal.pone.0046519 (PMC3465327; doi:10.1371/journal.pone.0046519)
Supplement: Table S1 — Case and control repartition by region. (DOCX) [file pone.0046519.s009.docx]

| **Region** | **Geographical Location** | **Controls** | **Cases** | **Total** |
| --- | --- | --- | --- | --- |
| NA | -- | 0 | 1 | 1 |
| 1 | Southeastern | 275 | 39 | 314 |
| 2 | Southern | 223 | 350 | 573 |
| 3 | London | 198 | 183 | 381 |
| 4 | Eastern | 338 | 498 | 836 |
| 5 | Northwestern | 314 | 55 | 369 |
| 6 | North Midland | 138 | 99 | 237 |
| 7 | Southwestern | 247 | 371 | 618 |
| 8 | Midlands | 317 | 16 | 333 |
| 9 | Northern | 262 | 292 | 554 |
| 10 | East and West Ridings | 207 | 7 | 214 |
| 11 | Wales | 146 | 10 | 156 |
| 12 | Scotland | 273 | 3 | 276 |
| Total | -- | 2938 | 1924 | 4862 |
