# Supplementary material for: Rare and Low Frequency Variant Stratification in the UK Population: Description and Impact on Association Tests
Source: PLoS One. 2012 Oct 5;7(10):e46519. doi: 10.1371/journal.pone.0046519 (PMC3465327; doi:10.1371/journal.pone.0046519)
Supplement: Table S2 — Description of the “others” SNPs according to the number of minor allele copies in the different samples. (DOCX) [file pone.0046519.s010.docx]

| **58BC** | **UKBS** | **T2D** | **# of SNPs** |
| --- | --- | --- | --- |
| 0 | 1 or 2 | 0 | 2,372 |
| 1 or 2 | 0 | 0 | 2,502 |
| 1 | 1 | 0 | 691 |
| 0 | 0 | 1 or more | 4,230 |
| 0 | 1 or 2 | 1 or more | 2,436 |
| 1 or 2 |  | 1 or more | 2,576 |
| 1 | 1 | 1 or more | 1,1283 |
