# Supplementary material for: Rare and Low Frequency Variant Stratification in the UK Population: Description and Impact on Association Tests
Source: PLoS One. 2012 Oct 5;7(10):e46519. doi: 10.1371/journal.pone.0046519 (PMC3465327; doi:10.1371/journal.pone.0046519)
Supplement: Table S3 — Results of homogeneity tests performed to compare allele counts in the two subgroups of controls. Genomic Control lambda values (λGC) are provided for the different MAF categories. (DOCX) [file pone.0046519.s011.docx]

| **MAF Class** | **λ_GC_** |
| --- | --- |
| Common | 1.006 |
| Low Freq | 1.021 |
| Rare | 1.081 |
| "others" | 2.145 |
