# Supplementary material for: Rare and Low Frequency Variant Stratification in the UK Population: Description and Impact on Association Tests
Source: PLoS One. 2012 Oct 5;7(10):e46519. doi: 10.1371/journal.pone.0046519 (PMC3465327; doi:10.1371/journal.pone.0046519)
Supplement: Table S4 — Number and proportion of variants that differ the most (p-value<5.10−4) between the 12 UK regions in the different MAF categories. (DOCX) [file pone.0046519.s012.docx]

| **MAF Class** | **# of SNPs** | **Proportion** |
| --- | --- | --- |
| Common | 216 | 0.08 |
| LowFreq | 17 | 0.06 |
| Rare | 16 | 0.08 |
| “others” | 0 | 0 |
