# Supplementary material for: Rare and Low Frequency Variant Stratification in the UK Population: Description and Impact on Association Tests
Source: PLoS One. 2012 Oct 5;7(10):e46519. doi: 10.1371/journal.pone.0046519 (PMC3465327; doi:10.1371/journal.pone.0046519)
Supplement: Table S5 — Repartition of the SNPs seen in only one region in each dataset, UKBS and 58BC. SNPs unique to each of the 12 regions are compared for their regional distribution in the two datasets and those found in the same region in the two datasets are highlighted. (DOCX) [file pone.0046519.s013.docx]

|  |  | **Regions UKBS** | | | | | | | | | | | |  | |  |
| --- | --- | --- | --- | --- | --- | --- | --- | --- | --- | --- | --- | --- | --- | --- | --- | --- |
|  |  | **1** | **2** | **3** | **4** | **5** | **6** | **7** | **8** | **9** | **10** | **11** | **12** | | ***Total*** | |
| **Regions 58BC** | **1** | 1 | 1 | 3 | 1 | 1 | 0 | 1 | 0 | 4 | 2 | 0 | 0 | | *14* | |
|  | **2** | 4 | 1 | 3 | 4 | 0 | 0 | 0 | 0 | 2 | 0 | 0 | 0 | | *14* | |
|  | **3** | 2 | 2 | 2 | 2 | 1 | 0 | 1 | 5 | 2 | 2 | 0 | 1 | | *20* | |
|  | **4** | 5 | 4 | 3 | 7 | 2 | 0 | 3 | 1 | 7 | 3 | 2 | 2 | | *39* | |
|  | **5** | 1 | 0 | 4 | 2 | 5 | 0 | 3 | 3 | 0 | 0 | 0 | 2 | | *20* | |
|  | **6** | 2 | 0 | 2 | 2 | 2 | 0 | 1 | 3 | 4 | 0 | 1 | 0 | | *17* | |
|  | **7** | 7 | 6 | 1 | 1 | 3 | 0 | 0 | 0 | 1 | 0 | 0 | 1 | | *20* | |
|  | **8** | 2 | 3 | 1 | 3 | 4 | 0 | 1 | 1 | 3 | 1 | 1 | 1 | | *21* | |
|  | **9** | 1 | 0 | 4 | 1 | 3 | 0 | 1 | 2 | 1 | 0 | 0 | 1 | | *14* | |
|  | **10** | 1 | 0 | 1 | 2 | 4 | 0 | 0 | 3 | 2 | 0 | 0 | 0 | | *13* | |
|  | **11** | 2 | 1 | 1 | 1 | 4 | 0 | 1 | 1 | 0 | 0 | 0 | 1 | | *12* | |
|  | **12** | 2 | 1 | 0 | 1 | 2 | 1 | 5 | 2 | 2 | 0 | 0 | 0 | | *16* | |
|  | ***Total*** | *30* | *19* | *25* | *27* | *31* | *1* | *17* | *21* | *28* | *8* | *4* | *9* | | *220* | |
