# Supplementary material for: Rare and Low Frequency Variant Stratification in the UK Population: Description and Impact on Association Tests
Source: PLoS One. 2012 Oct 5;7(10):e46519. doi: 10.1371/journal.pone.0046519 (PMC3465327; doi:10.1371/journal.pone.0046519)
Supplement: Table S6 — P-values of the ANOVA comparing the values of the different PCs over the 12 region. (DOCX) [file pone.0046519.s014.docx]

| **Set of variants^a^** | **Common** | **LowFreq** | **Rare** |
| --- | --- | --- | --- |
| **PC1** | 9.66 10^-12^ | <2 10^-16^ | 0.55 |
| **PC2** | <2 10^-16^ | 0.27 | 4.37 10^-3^ |
| **PC3** | <2 10^-16^ | 5.33 10^-6^ | 4.95 10^-6^ |
| **PC4** | 0.08 | 0.05 | 0.39 |
| **PC5** | 0.20 | 0.28 | 0.03 |
| **PC6** | 0.04 | 0.46 | 0.64 |
| **PC7** | 0.00 | 0.00 | 0.63 |
| **PC8** | 0.71 | 0.33 | 0.42 |
| **PC9** | 0.43 | 0.00 | 0.98 |
| **PC10** | 0.54 | 0.00 | 0.10 |

^a^ Set of variants considered in the PCA (Common, Low Frequency or Rare)
