# Supplementary material for: Rare and Low Frequency Variant Stratification in the UK Population: Description and Impact on Association Tests
Source: PLoS One. 2012 Oct 5;7(10):e46519. doi: 10.1371/journal.pone.0046519 (PMC3465327; doi:10.1371/journal.pone.0046519)
Supplement: Table S7 — Correlation (R2 values) between the first two PCs (PC1.x and PC2.x) obtained on the four disjoint subset x (x = 1 to 4) of 11,848 common variants. The last three columns Top10.x give the cumulative R2 values over the top 10 PCs to show how each PC in line is capture by the combined top 10 PC of the different subsets. (DOCX) [file pone.0046519.s015.docx]

|  | **PC1.1** | **PC2.1** | **PC1.2** | **PC2.2** | **PC1.3** | **PC2.3** | **PC1.4** | **PC2.4** | **Top10.1** | **Top10.2** | **Top10.3** | **Top10.4** |
| --- | --- | --- | --- | --- | --- | --- | --- | --- | --- | --- | --- | --- |
| **PC1.1** | 1.00 | 0.00 | 0.26 | 0.09 | 0.33 | 0.01 | 0.33 | 0.00 | 1.00 | 0.36 | 0.35 | 0.33 |
| **PC2.1** |  | 1.00 | 0.07 | 0.23 | 0.01 | 0.28 | 0.00 | 0.29 | 1.00 | 0.30 | 0.29 | 0.30 |
| **PC1.2** |  |  | 1.00 | 0.00 | 0.21 | 0.13 | 0.28 | 0.06 | 0.33 | 1.00 | 0.35 | 0.35 |
| **PC2.2** |  |  |  | 1.00 | 0.14 | 0.18 | 0.06 | 0.26 | 0.32 | 1.00 | 0.33 | 0.33 |
| **PC1.3** |  |  |  |  | 1.00 | 0.00 | 0.28 | 0.02 | 0.35 | 0.36 | 1.00 | 0.31 |
| **PC2.3** |  |  |  |  |  | 1.00 | 0.02 | 0.26 | 0.29 | 0.32 | 1.00 | 0.29 |
| **PC1.4** |  |  |  |  |  |  | 1.00 | 0.00 | 0.33 | 0.35 | 0.31 | 1.00 |
| **PC2.4** |  |  |  |  |  |  |  | 1.00 | 0.30 | 0.32 | 0.29 | 1.00 |
