# Supplementary material for: Rare and Low Frequency Variant Stratification in the UK Population: Description and Impact on Association Tests
Source: PLoS One. 2012 Oct 5;7(10):e46519. doi: 10.1371/journal.pone.0046519 (PMC3465327; doi:10.1371/journal.pone.0046519)
Supplement: Table S8 — Genomic control coefficient lambda λGC obtained for the different tests of association performed on the type 2 diabetes dataset with the different sets of variants. (DOCX) [file pone.0046519.s016.docx]

|  |  |  |  |  | **Within the Rare set** | |
| --- | --- | --- | --- | --- | --- | --- |
|  | **Common** | **LowFreq** | **Rare** |  | **MAF<=0.005** | **MAF>0.005** |
| **CMH^a^** | 0.993 | 1.023 | 1.802 |  | 1.802 | 1.103 |
| **Raw^b^** | 1.006 | 1.058 | 2.193 |  | 2.193 | 1.121 |
| **common.2^c^** | 0.993 | 1.042 | 2.072 |  | 2.137 | 1.067 |
| **common.3** | 0.993 | 1.042 | 2.052 |  | 2.129 | 1.066 |
| **common.4** | 0.990 | 1.048 | 1.927 |  | 2.053 | 1.104 |
| **common.10** | 0.978 | 1.027 | 1.675 |  | 1.880 | 1.100 |
| **lowfreq.2** | 0.995 | 1.044 | 1.969 |  | 2.085 | 1.072 |
| **lowfreq.3** | 0.995 | 1.041 | 1.945 |  | 2.044 | 1.064 |
| **lowfreq.4** | 0.992 | 1.033 | 1.922 |  | 2.022 | 1.071 |
| **lowfreq.10** | 0.980 | 1.012 | 1.767 |  | 1.931 | 1.112 |
| **rare.2** | 0.993 | 1.040 | 2.085 |  | 2.152 | 1.089 |
| **rare.3** | 0.991 | 1.043 | 2.081 |  | 2.143 | 1.090 |
| **rare.4** | 0.989 | 1.032 | 1.960 |  | 2.074 | 1.081 |
| **rare.10** | 0.974 | 1.014 | 1.858 |  | 2.013 | 1.072 |
| **EMMAX.common^d^** | 1.001 | 1.052 | 2.182 |  | 2.182 | 1.115 |
| **EMMAX.lowfreq** | 1.001 | 1.052 | 2.182 |  | 2.182 | 1.115 |
| **EMMAX.rare** | 1.000 | 1.048 | 2.165 |  | 2.180 | 1.114 |

^a^ Cochran-Mantel-Haenszel test accounting for the 2 different regions

^b^ Test not corrected for population stratification

^c^ Test corrected for population stratification using different number of PCs computed on different pruned MAF sets (i.e.; common.2 means that the PCs were computed on the common varint sets and 2 the test is adjusted on 2 such PCs).

^d^ Test performed using the mixed model implemented in EMMAX with the relatedness matrix computed either on the common, low frequency or rare variant sets.
